# Insights on the Study of Nafion Nanoscale Morphology by Transmission Electron Microscopy

**DOI:** 10.3390/membranes3040424

**Published:** 2013-12-16

**Authors:** Sergey Yakovlev, Nitash P. Balsara, Kenneth H. Downing

**Affiliations:** 1 Materials Sciences Division, Lawrence Berkeley National Laboratory, 1 Cyclotron Road, Berkeley, CA 94720, USA; E-Mail: npbalsara@lbl.gov; 2 Department of Chemical and Biomolecular Engineering, University of California, Berkeley, CA 94720, USA; 3 Life Sciences Division, Lawrence Berkeley National Laboratory, 1 Cyclotron Road, Berkeley, CA 94720, USA; E-Mail: khdowning@lbl.gov

**Keywords:** Nafion, electron microscopy, electron beam damage

## Abstract

Nafion is one of the most common materials used for polyelectrolyte membranes and is the standard to which novel materials are compared. In spite of great interest in Nafion’s nanostructure, it is still a subject of controversy. While multiple research efforts have addressed Nafion’s morphology with Transmission Electron Microscopy, the results of these efforts have often been inconsistent and cannot satisfactorily describe the membrane structure. One of the reasons for differences in the reported results is the lack of sufficient control over the damage caused by electron beam irradiation. In this work, we describe some aspects of damage in the material that have a strong influence on the results. We show that irradiation causes mass loss and phase separation in the material and that the morphologies that have been observed are, in many cases, artifacts caused by damage. We study the effect of the sample temperature on damage and show that, while working at low temperature does not prevent damage and mass loss, it slows formation of damage-induced artifacts to the point where informative low-dose images of almost undamaged material may be collected. We find that charging of the sample has a substantial effect on the damage, and the importance of charge neutralization under irradiation is also seen by the large reduction of beam induced movement with the use of an objective aperture or a conductive support film. To help interpret the low-dose images, we can apply slightly higher exposures to etch away the hydrophobic phase with the electron beam and reveal the network formed by the hydrophilic phase. Energy loss spectroscopy shows evidence that fluorine removal governs the beam damage process.

## 1. Introduction

Nafion is a Perfluorosulfonic Acid (PFSA) membrane material that is widely used and has considerable importance for ongoing developments in energy-related technologies. Since its discovery by DuPont, a great deal of effort has been spent to understand the mechanisms of its function in charge transport [1,2]. In spite of these efforts, many questions remain unanswered.

In early studies of ionomers, mechanical properties were found to be influenced by clustering of ionic species that create crosslinks between the polymer chains and affect their mobility. Later it was suggested that clusters also play a major role in shaping continuous pathways for proton transfer through the membrane. Within this model, having well-connected clusters is important to provide continuous ion flow. Thus, the geometry of the clusters may be a key factor affecting membrane performance. Unfortunately, due to the small size of the clusters, imaging their geometry has proven to be difficult.

Most of the available information about the structure of Nafion comes from the results of X-ray and neutron scattering experiments that cannot uniquely identify the structure in direct space. There have been considerable efforts to visualize Nafion’s morphology in direct space by electron microscopy (EM), but results have not always been consistent. For example, it has been reported that there were no clusters visible in unstained material although they could be visualized by certain types of staining [3]. In other work even untreated material has shown distinguishable cluster-like morphology [4]. Clusters have been reported to grow larger under irradiation [5], which suggests that the observed features are generated by radiation damage. Marking the ionic sites in some ionomers with counterions also has shown contradictory effects—in some cases, increasing contrast and in others not [6]. While counterions in Nafion typically increase contrast, the nature of this contrast has not been convincingly explained. Because of the insufficient control over experimental parameters in much of the previously published work and the large number of controversial and inconsistent measurements, we were prompted to investigate the reasons for these discrepancies and possibilities of resolving the real morphology.

Direct comparison of the different EM experiments on Nafion is difficult because most reported work did not address the problem of radiation sensitivity. Damage of polytetrafluoroethylene (Teflon)—the backbone of the Nafion molecule—has been shown to be very extensive [7], similar to other fluorinated compounds [8]. The high sensitivity of Teflon implies that the irradiation sensitivity of Nafion should also be substantial. Still, no comprehensive study of its electron beam sensitivity under irradiation in TEM has been presented. In this work, we attempt to fill this gap and to study the effects irradiation has on our ability to image Nafion’s structure.

## 2. Results and Discussion

### 2.1. Results

A striking observation on Nafion films in the electron microscope is that electron beam irradiation produces damage that strongly depends on the dose rate at least as much as on the total exposure. This behavior is particularly noticeable with films thin enough to be useful for high resolution structural studies, less than around 50 nm. At dose rates over 10,000 e/nm^2^ s, mass loss in these films is so rapid and extreme that the electron beam physically destroys the material and makes a hole in the film. Reducing the dose rate reduces the mass loss significantly, allowing the collection of images. Similar effects have been seen in other radiation-sensitive specimens such as hydrated proteins [9], but not nearly to the extent as with Nafion. As with some other specimens, charging is also a significant problem, but again the situation with Nafion is much worse. Using an objective aperture to reduce charging effects is extremely important for any experiments on Nafion since without this aperture, even low irradiation doses cause large movement of the sample under the beam. Still, even with this aperture, irradiation causes changes within the Nafion film that can be observed as movement of features from the center of the irradiated area to the periphery. This movement causes defects in the image that appear similar to drift or astigmatism, but directed out of the center of the image. Using extremely low dose rates (below 500 e/nm^2^ s) minimizes and sometimes completely eliminates these movements. The exact behavior depends on the experimental parameters, most importantly the temperature of the sample but also on the state of the material (acidic or ion exchanged). Below we will discuss these effects starting with the temperature dependence of acidic Nafion.

At room temperature irradiation of thin Nafion films with a low dose rate around 500 e/nm^2^ s causes a slow decrease in mass thickness. This change can be seen qualitatively in the image intensity, and can be quantified as mass loss which is characterized electron energy loss spectroscopy (EELS) as described below. The thickness decreases exponentially, stabilizing to some equilibrium value which depends on the exact irradiation dose rate, with higher dose rates resulting in lower equilibrium thickness. At any moment the thickness within the exposed area is almost uniform and with time develops a sharp step at the edge of the irradiated area. The sensitivity of the sample to irradiation decreases as damage accumulates. Films that have reached a stable thickness appear very stable to irradiation and dose rates of 10,000 e/nm^2^ s may be easily applied without causing any further, noticeable mass loss.

**Figure 1:**
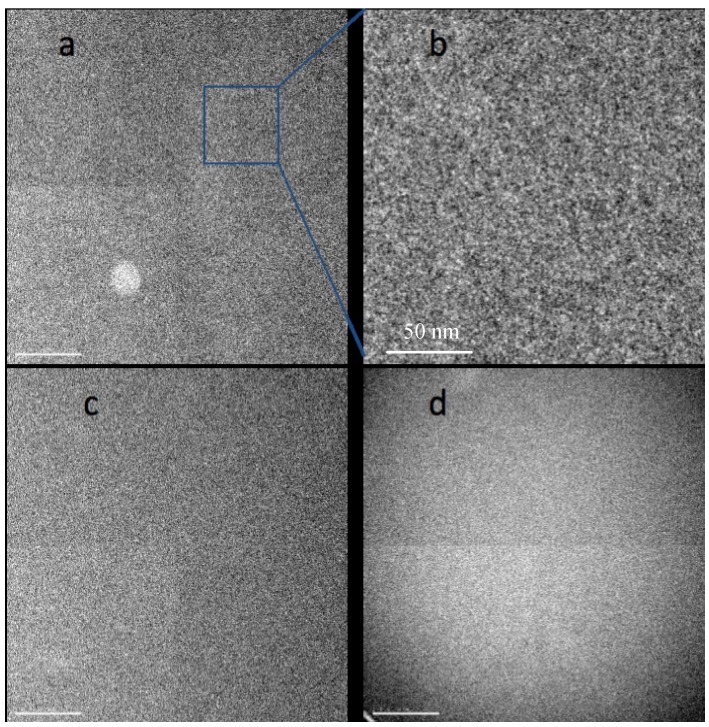
Bright field TEM images of acidic Nafion films initially about 50 nm thick, suspended over a hole in the support film, recorded at room temperature with 200 kV beam, 500 e/nm^2^ at rate 500 e/nm^2^ s. (**a**) from initially undamaged sample; (**b**) section magnified 4×; (**c**) same area after exposure to 10,000 e/nm^2^; (**d**) image of specimen at −180 °C recorded with 300 e/nm^2^ at rate 300 e/nm^2^ s, after pre-exposure to 5000 e/nm^2^. Differences in image quadrants arise from variations in gain correction for the camera used in recording these images. Scale bars in **a**, **c** and **d** are 200 nm; in **b** 50 nm. A more complete set of images is in Supplementary Figure S1.

Figure 1a,b shows an image of a Nafion film around 35 nm thick obtained in bright field TEM mode with 500 e/nm^2^ exposure at a dose rate 500 e/nm^2^ s. We have found that recording images in focus with a small objective aperture (corresponding to a cutoff at about 0.5 nm) maximizes the contrast of heavy atom clusters by reducing the phase contrast granularity usually associated with high resolution TEM imaging. The image shows a relatively low level of contrast and high level of noise due to the low dose and in-focus conditions. It is still possible to conclude that the morphology of the sample is not uniform but appears as densely packed, disconnected clusters (dark spots) with irregular shape and highly variable sizes, in the 1–3 nm range. Changes in the appearance of these features with defocus confirm that they are real rather than simply shot noise. However, the noise level in images recorded with exposures low enough to avoid serious damage makes it difficult to derive a satisfying characterization of the morphology. Continued irradiation of the sample brings some rearrangement to the structure. In subsequent images some of the clusters lose contrast while others appear and become darker. An image of the same area collected after an accumulated exposure of 10,000 e/nm^2^ is shown in Figure 1c. After such a dose the sample thickness has already stabilized at a value about 80% of the initial thickness. Now we can see a slightly higher level of contrast and larger clusters than before irradiation. However, the images are still dominated by shot noise so that assessing changes in the sample morphology is difficult. 

One observation we have made comparing images of Nafion films before and after irradiation is that large features that are sometimes observed in as-cast films frequently disappear with irradiation. For example Figure 1a shows bright a spot with a diameter about 100 nm in the bottom left quadrant that is common with cast films; this spot is not seen in Figure 1c. The most probable explanation is that this feature is an area with lower thickness formed during casting. Irradiation softens the material and helps to smooth any surface roughness that was present before irradiation. Surface tension may be a primary driving force for smoothing such surface roughness.

Continued irradiation of a stabilized Nafion film slowly introduces effects that have the appearance of phase separation. We have seen various artifacts that have no relationship to the morphology of virgin material. Figure S2 shows one example that looks like a highly phase separated material with features of an interconnected network. This particular sample was pre-irradiated with a low exposure rate and then exposed to a dose rate of 10,000 e/nm^2^ s for one minute. What distinguishes such artifacts is a length scale larger than that of phase separations observed in scattering experiments. Imaging conditions that produce such artifacts have to be avoided.

Cooling the sample to liquid nitrogen temperature affects its response to the interaction with the electron beam. While low temperature certainly reduces some effects of irradiation by suppressing diffusion of products of radiolysis, we find that it also hinders reaching a stable thickness and equilibrium chemical composition in the Nafion samples. In such samples we observe slower but more constant mass loss without reaching an equilibrium thickness. The thickness loss is typically greater in the center of the irradiated area, causing formation of a meniscus type surface. The collective movement of the features from the center to periphery is still seen at even lower intensity than at room temperature, which prompted us to use a lower dose rate of 300 e/nm^2^ s for cryo-imaging.

The appearance of the image with cryo TEM observation (−180 °C) and exposures of 300 e/nm^2^ is very similar to that seen at room temperature, with small clusters visible just above the noise level (see Figure S1). While continued irradiation changes the shapes and positions of the clusters randomly, a general shift of features from center to periphery may also be observed. After a dose of 5000 e/nm^2^, the clusters are slightly bigger and more pronounced. The meniscus shape of the sample may be clearly observed (Figure 1d). After bringing the exposure to 10,000 e/nm^2^ with the same dose rate, we observe formation of a hole in the center of the image that eventually expands to fill the entire irradiated area.

**Figure 2:**
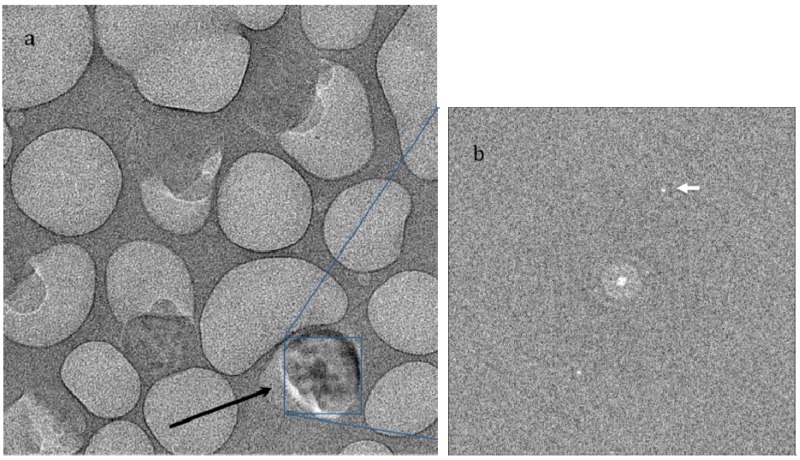
(**a**) Image of a thick film exposed to 1000 e/nm^2^ at 300 kV, showing gas evolution under the beam irradiation. Black arrow shows a single crystal of polytetrafluoroethylene that formed inside a bubble during exposure. (**b**) Fourier transform of boxed area indicating peaks (white arrow) corresponding to the PTFE spacing of 5.7 Å, which are only visible if the transform is taken from area including crystal.

As we see from our experiments, some mass loss is always observed in Nafion and needs to be recognized when images are interpreted. To obtain better insight on the mass loss, we have examined thicker parts of the Nafion samples. In experiments with thicker films, on the order of 500 nm thick, we used a higher accelerating voltage of 300 keV to take advantage of the longer electron mean free path. We found that mass loss in Nafion is associated with formation of gas that sometimes creates bubbles. In thinner regions bubbles are never observed, even when much higher dose rate are used, presumably because the gas diffuses to the surface and escapes to the vacuum. In thick parts of the sample even low dose rates of 300 e/nm^2^ s at an accelerating voltage of 300 keV are sufficient for bubble formation. Bubbles appear and grow in size and number with irradiation. Figure 2a shows an image of a sample with gas bubbles after irradiation with an exposure of about 1000 e/nm^2^. One notable feature in Figure 2a is a single crystal, marked with an arrow, which grew during the period of bubble growth. The crystalline nature is clear from diffraction peaks visible in the Fourier transform of the image. Such crystalline diffraction is never observed with undamaged material. Presumably, high pressure inside the gas bubble creates conditions for nucleation and growth of single crystals of polytetrafluoroethylene. With continued irradiation, the crystals eventually disappear, as well as the bubbles.

**Figure 3:**
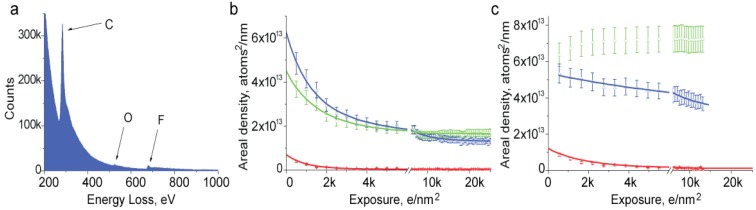
(**a**) Electron energy loss spectroscopy (EELS) spectrum collected from an acidic film of Nafion; (**b**,**c**) Changes in elemental composition of Nafion film with irradiation: (**b**) film suspended over holey support film; (**c**) film on continuous carbon support. Arrows indicate peaks and curves corresponding to elements: C—carbon (green), O—oxygen (red), F—fluorine (blue).

We have studied mass loss from Nafion by EELS to gain further insight on the effects of beam damage. We set the electron beam to irradiate a relatively large area of the sample and record a series of EELS spectra with accumulating total exposure. A spectrum collected from initially undamaged material is shown in Figure 3a. This spectrum was collected with parallel irradiation using a 500 e/nm^2^ exposure, collection time 2 s and detector collection semi angle 2.5 mrad. Energy dispersion on the spectrometer was set to 0.5 eV. Edges corresponding to carbon, oxygen and fluorine are visible at energies of 284, 532 and 685 eV. We used those edges to find the composition of the sample. Quantification was done using standard routines in Gatan Digital Micrograph software using a power law for background subtraction and the Hartree-Slater method for cross-section calculation.

Irradiation causes changes in the magnitude of peaks that reflect loss of elements from the sample. We found that elemental loss depends on many parameters including beam intensity, the initial thickness of the sample, the state of ion exchange and temperature. Consistent with the thickness change observed in Bright Field TEM observations, EELS experiments show that cryo-temperature somewhat slows down the elemental loss. It also makes elemental loss less selective, with fluorine and carbon loss more closely corresponding to their atomic composition. For this reason, along with the fact that the specimen thickness becomes non-uniform at low temperature, room temperature elemental loss appears more informative for understanding the final products of beam damage.

Figure 3b shows elemental loss from acidic Nafion at room temperature at a dose rate of 100 e/nm^2^ s. The very first experimental point shows that the fluorine content is about 1.3 times the carbon content. This indicates that some composition change has already happened since this ratio should be close to 2 for Nafion [2]. While in other experiments we were able to obtain a better match in composition by broadening the beam and reducing the dose, in this work conditions were chosen to mimic electron microcopy imaging of Nafion. We fit the experimental points with a single exponential curve. With this specimen, the best fit for carbon and fluorine is obtained with decay constants of 1711 ± 276 and 1676 ± 165 e/nm^2^, respectively. Loss of oxygen is faster and is well described by a decay constant of 811 ± 109 e/nm^2^. It is also evident that the final oxygen content in the sample is essentially zero while both carbon and fluorine come to some equilibrium value. Rapid removal of the oxygen is not surprising since the degradation of Nafion molecule was shown to start from the sulfuric acid group and ether side chain where all the oxygen is localized [10].

A more careful look into the data reveals that the carbon content, after reaching a minimum value, shows a weak increase, which apparently is a result of accumulation of radiation-induced contamination. The contamination is more evident with Nafion samples deposited on a continuous carbon support film. Irradiation of such samples causes an increase of the carbon content that begins with even relatively low exposures (Figure 3c). The strong rate of contamination suggests that it is not related to the vacuum conditions in the microscope but rather to surface diffusion of carbon along the Nafion surface.

The removal of fluorine and oxygen from samples on a continuous support film occurs with lower rates than with unsupported films, with time constants of 3400 and 1600 e/nm^2^, respectively, for the film in Figure 3c. It is also noteworthy that total loss of fluorine at the point where the sample reaches an equilibrium concentration is significantly lower for a sample on a continuous support compared to a film suspended in vacuum. This observation provides additional evidence that charging of Nafion may be a major process causing damage. Using a conductive support films is well known to be one of the best solutions to the problem of charging.

One further EELS observation worthy of mention is a strong dependence of the elemental loss on the thickness of the sample. Thick samples generally show substantially higher fluorine content which decreases more slowly. In very thick samples fluorine content remains high and never approaches the carbon content.

A large number of reports have described electron microscopy studies of Nafion membranes following ion exchange [3,4,5,11,12,13,14]. Ion exchange has long been recognized as a good approach for increasing the contrast in Nafion samples and for marking the hydrophilic domains in the material. We have repeated such experiments to examine the difference between acidic and ion exchanged material. While in various experiments a large number of different counterions have been used, we have focused on copper and lead ions as two of the most common. Dramatic effects are seen in images that depend on the evolution of the chemical composition with irradiation.

Adding counterions to the Nafion film has an effect on reducing mass loss similar to (although smaller in magnitude) the effect of using a conductive support film (not shown). We measured the concentration of copper by EELS and found it to be unaffected by irradiation. The loss of the other elements thus results in enriching the sample with copper.

**Figure 4:**
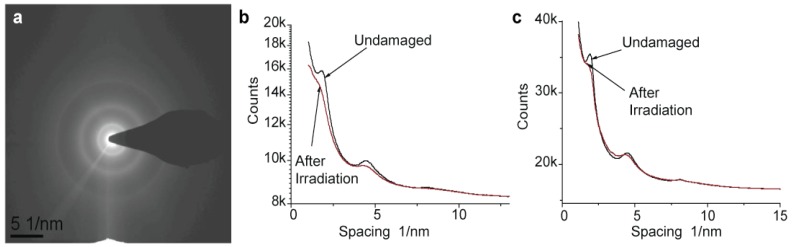
Electron diffraction from Nafion. (**a**) Diffraction pattern collected from a nearly undamaged Nafion film (200 kV electrons; dose used for data collection was 30 e/nm^2^, dose rate 15 e/nm^2^ s); (**b**,**c**) profiles of diffraction pattern collected from initially undamaged film (black lines) and films pre-irradiated with 1000 e/nm^2^ (red lines): (**b**) room temperature and (**c**) −180 °C.

In determining an exposure limit for imaging sensitive samples, the mass loss is not necessarily the best criterion, as some damage to the structure may occur without mass loss. A common way to determine an exposure limit for crystalline specimens is the observation of changes in an electron diffraction pattern. Because Nafion is a semi-crystalline polymer with some segments of the polytetrafluoroethylene backbones forming crystals, observation of changes in diffraction patterns may be informative. We have studied changes in diffraction patterns at both room and cryo temperature and found that temperature has only a subtle effect on the diffraction pattern decay. Figure 4a shows a diffraction pattern collected from an initially undamaged Nafion film using a very low exposure of 30 e/nm^2^ and dose rate of 15 e/nm^2^ s. The outer ring that corresponds to a spacing of 1.2 Å (002) appears relatively sharp, the second ring at a spacing of about 2 Å (101) is more diffuse, and the inner ring at 5.6 Å (101) is relatively sharp.

In order to illustrate the pattern decay quantitatively, Figure 4b,c shows profiles of diffraction patterns collected at room and cryo temperatures at a low exposure of 30 e/nm^2^, *i.e*., from nearly undamaged material, and after irradiation with 1000 e/nm^2^. Rings clearly visible at the beginning of the exposure decay with irradiation of 1000 e/nm^2^. After an exposure of 3000 e/nm^2^ the rings at 1.2 Å and 5.6 Å completely disappear. In some cryo samples, however, mass loss is so strong that we observed the formation of holes in the sample even before complete decay of the diffraction pattern. A slight increase in the dose rate seems to increase the pattern decay rate linearly while application of a dose rate over 1000 e/nm^2^ s causes changes so rapid that no quantitative information could be obtained.

We were not able to observe any of the sharp features in Fourier transforms of minimally damaged Nafion images, consistent with the notion that charging and damage cause significant movement of both the specimen and image. However, crystals that grow inside the gas bubbles under irradiation in thick areas (Figure 2) produce peaks in the image transforms at a spacing of 5.6 Å, similar to the spacing of the inner diffraction ring of Nafion. This reflection may be easily observed in electron diffraction patterns from the thicker parts of the sample but only after some initial irradiation. It is important to note here that all crystalline reflections induced by irradiation are very sharp, indicating good packing of atoms in a crystal lattice, in contrast to the weak rings observed from Nafion under low dose irradiation. All the features we observe in diffraction patterns are characteristics of the polytetrafluoroethylene crystals rather than specific to the ionic sites in ionomers. A peak at 5 nm has been seen in X-ray diffraction of bulk Nafion, but we were not able to see this peak with thin membranes of either hydrated or dry samples.

We found that films doped with counterions are much more stable to irradiation. While the initial behavior is similar to that of undoped films, doped samples show less beam-induced motion and come to equilibrium thickness much faster and with less final mass loss than acidic samples. We interpret at least part of this difference to increased electrical conductivity, especially as the relative copper concentration increases. Low dose images of undamaged doped Nafion collected with a dose rate below 500 e/nm^2^ s (not shown here) appear to be very similar to images of acidic Nafion obtained under the same conditions, with no obvious contrast from atomic clusters. At a dose rate of about 1000 e/nm^2^ s even at relatively low total exposures, we start to observe differences between the acidic and doped samples that are related to the mechanism of damage. At exposures over 1000 e/nm^2^, the mass loss is already noticeable, causing enrichment of the sample with the metal (because neither lead nor copper leave the sample under irradiation). This process is similar to the concentration of sulfur in an acidic sample, but different in that metal-rich samples have higher conductivity and can tolerate significantly higher doses without physical destruction of the film.

**Figure 5:**
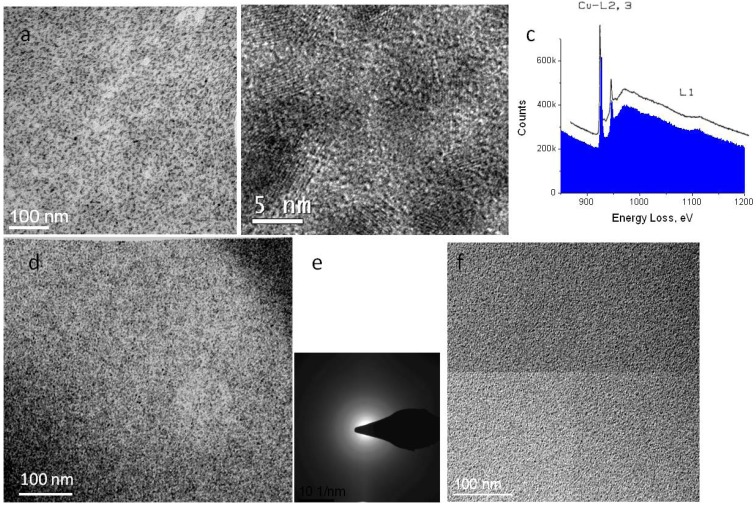
Precipitates in doped Nafion membrane. (**a**,**b**) Cu-treated membrane irradiated at room temperature; (**c**) EELS spectrum (blue) showing edge corresponding to L edge of copper oxide (superimposed curve); (**d**,**e**) cryo-image of Cu-treated Nafion and corresponding electron diffraction pattern; (**f**) Pb-treated Nafion.

Precipitation of the heavy elements following exposure may be observed in electron micrographs. In the case of copper, precipitates created with dose rates over 1000 e/nm^2^ s are extremely well defined (Figure 5a). In fact, high resolution images reveal that precipitates are nano-sized crystals (Figure 5b). Because the film is no longer sensitive to irradiation, very high doses may be applied to analyze the crystal composition. EELS analysis reveals a spectrum near the copper L edge that is identical to that observed from copper oxide (Figure 5c) [15]. By electron tomography, we have seen that the precipitates are distributed throughout the thickness of the sample, confirming that their development is not a surface-related phenomenon.

At cryo-temperatures, growth of precipitates may be observed somewhat similar to room temperature (Figure 5d). However, electron diffraction reveals that precipitates of copper formed at cryo-temperature are amorphous, rather than crystalline (Figure 5e). Whether precipitates crystallize or not, heavy counterions have a protective effect against irradiation by reducing the mass loss. Formation of a hole in the film under irradiation is generally not observed with doped samples. In contrast to copper, ion exchange with lead did not produce crystalline precipitates. At room as well as at cryo-temperature, such films showed only amorphous precipitates (Figure 5f).

**Figure 6:**
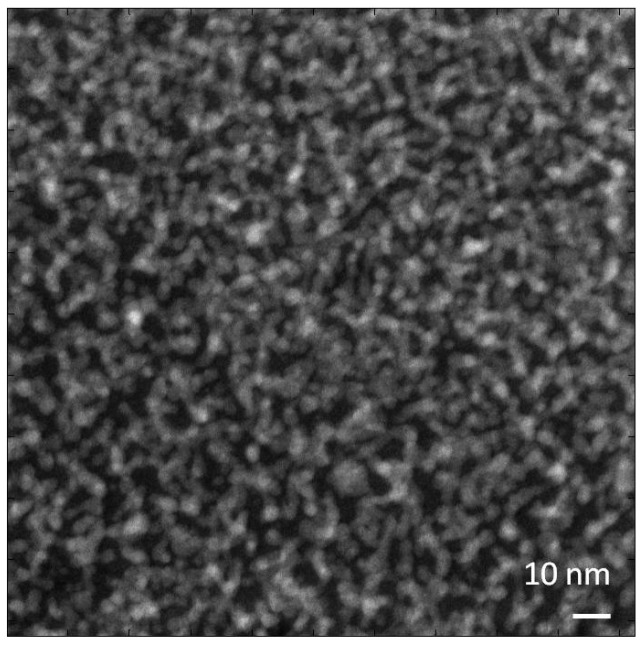
HAADF image of acidic Nafion membrane. Exposure used for the imaging was 10,000 e/nm^2^ with a 300 kV beam.

Observations of Nafion in the HAADF STEM mode produce high-contrast artifacts. Figure 6 shows an example of such an image. This kind of image is typical of even the very first scan, which generally requires an exposure of about 10,000 e/nm^2^. Reducing the dose to 1000 e/nm^2^ produces similar images but with higher noise and much lower levels of contrast. It is important to note that the contrast development is associated with thickness loss in the film. We believe that the contrast in the image presented in Figure 6 is in large part a consequence of preferential etching of the tetrafluoroethylene phase [16].

**Figure 7:**
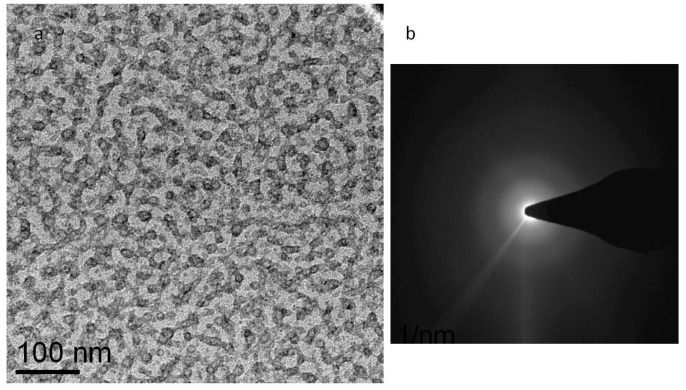
Imaging of highly damaged, hydrated film. (**a**) TEM Bright Field image of hydrated Nafion and (**b**) corresponding electron diffraction pattern. Sample was exposed at a dose rate of 10,000 e/nm^2^ s to a total exposure about 50,000 e/nm^2^.

A final observation concerns damage to cryo-preserved Nafion saturated with water. Samples were saturated with water at room temperature and at 80 °C, corresponding to nominal water uptake of 20 and 35 percent, respectively [17]. Images of the undamaged material are very similar to the images collected from the dry samples, with a very low level of contrast and disordered features. We observed that saturation of Nafion with water substantially reduces the electron beam sensitivity and that this reduction seems to increase with increased water uptake. However, damage is still noticeable at any water uptake, and irradiation with dose rates higher than 1000 e/nm^2^ s for prolonged time generates apparent phase separation in the material (Figure 7). Low dose images appear very similar to the images of dry Nafion and only show noisy, disordered clusters with an average size of a few nm. Increasing the water content in Nafion does not visibly affect the observed morphology.

### 2.2. Discussion

In this work, we have shown that electron microscopy images obtained from Nafion cannot be interpreted without considering the damage of Nafion, and our conclusions may be extended to other PFSA membranes as well. Due to the high sensitivity of these materials, obtaining meaningful images showing the morphology of truly undamaged material is not possible, as the exposure required to produce a statistically well defined image is sufficient to cause noticeable damage. The key to understanding how to study sample morphology is the analysis of changes caused by damage. We have found that, at low irradiation dose rates and low exposures, changes are slow enough that features of the virgin material morphology can be seen, although they are quite noise-limited. An increase in dose rate causes rapid and significant changes in morphology, which may be one reason for variations in earlier published results. One important observation in our work that seems to contradict most earlier published results is that adding counterions to Nafion does not noticeably increase the contrast in EM imaging of undamaged material. We demonstrated that the increase in contrast seen earlier can be a consequence of radiation damage that should be avoided. We found that most of the observed features that are more than 5 nm in size are a result of beam damage, and should not be confused with the morphology of pristine Nafion.

Investigation of the morphology of pristine Nafion in 2D TEM Bright Field images (Figure 1) shows clusters with a characteristic size of a few nanometers and inter-cluster spacings close to 5 nm—the value also inferred from SAXS and SANS experiments. However, observing these spacings in 2D images is surprising as such images represent the projection of the structure and would thus be expected to have overlapping images of clusters distributed through the thickness of the sample. The thickness of our thinnest samples is about 35 nm, which should have caused multiple overlap of the cluster images and apparent decrease of inter-cluster distance in the 2D images. While this kind of problem is typically solved by electron tomography, for Nafion this is extremely challenging due to movement of the features under the beam.

To explain the observed morphology, we suggest that most of the hydrophilic regions do not produce enough contrast to be directly observed in the electron microscope. This is reasonable because scattering densities of the two phases of Nafion are similar: the ether side chains have lower local mass density than fluorocarbon backbones, but the presence of heavier sulfur atoms matches the contrast of the denser phase so that no features are visible. However, the morphology of the hydrophilic phase is apparently highly non-uniform with some occasional areas of high sulfur concentration, which are responsible for the observed cluster contrast. Clusters with smaller sizes are not visible at the low exposures necessary for avoiding damage but contribute to the data collected by SAXS. An alternative explanation would suggest that the features we observe result from multiple overlap of clusters that in a random fashion creates an appearance somewhat similar to an image of a single layer of clusters. In particular, the actual size of the features may be preserved in the images even with strong overlapping [18].

We have shown that electron beam damage in Nafion is associated with rapid gas formation. It is interesting that, in the thin samples, we never observe formation of bubbles, which is evidence that diffusion of the gas to the surface of the sample is fast. Formation of gas is consistent with previous studies in which the products of Teflon damage have been investigated by means of mass spectroscopy [7]. Among the gases produced by irradiation, a significant fraction were shown to be simple fluorocarbons. Our EELS elemental loss study shows that while the fluorine loss is faster than carbon loss, the time constant for loss of both elements is similar. This can be expected because pure carbon typically is not removed by irradiation in large quantities. In Nafion, carbon likely is removed only as a part of fluorocarbon molecules, and the loss of carbon is directly related to loss of fluorine. In contrast, fluorine may be removed as fluorine gas as well as part of a fluorocarbon molecule, e.g., tetrafluoromethane or tetrafluoroethylene.

Gases that are produced by irradiation and trapped in the bubbles sometimes cause the formation of single crystals that reveal diffraction peaks at 5.7 Å corresponding to diffraction from polytetrafluoroethylene, which has a monoclinic lattice with b spacing of 5.6 Å [19,20]. Crystals of polytetrafluoroethylene can be grown by initiated chemical vapor deposition [14]. Products of Nafion radiolysis in the electron microscope could serve as initiator and monomer supply for the reaction under electron beam irradiation.

Charging of Nafion under the electron beam may be the most important process influencing the damage. Some level of charging is always observed on all soft materials. Compared to charging in most hydrocarbons, though, this problem is much greater for Nafion because of its low conductivity that does not significantly increase with irradiation. In most hydrocarbons, electron irradiation causes loss of hydrogen, transforming the material to a carbon-rich and more conductive state. In Nafion, loss of fluorine mimics the hydrogen loss in hydrocarbons but is accompanied by the loss of carbon and therefore does not transform the material into a conductive phase as effectively. This is especially true at elevated irradiation dose rates. At low dose rates, fluorine loss is proportionately faster than carbon loss and a conductive state may be reached. Charging is evident, for example, from the behavior of the unscattered beam in diffraction mode. As the sample is moved, the shape of the beam changes and the beam moves around.

Another manifestation of charging is sputtering of small pieces of material from sharp edges of Nafion under the beam, which may be observed on the fluorescent screen. It is evident that such behavior is caused by excessive charge build-up on the tips of the sharp edges. Charging may also be the reason that the clusters and other features tend to move from the center to periphery of the irradiated areas, as observed at medium and elevated dose rates. The fact that the objective aperture reduces all of these effects supports the interpretation of their charge-induced character. While in acidic Nafion the mass loss at low dose rates causes enrichment of the material with carbon, making it somewhat more conductive, in samples doped with copper, this process is stronger and eventually transforms the film to a copper-rich conductive material that is stable at any beam intensity. Unfortunately, the counterions do not prevent changes in morphology that happen at the first moments of irradiation before the stable state is reached.

Using a conductive support film reduces the damage significantly, as we have observed in both diffraction and imaging modes. Unfortunately images of regular carbon support films are similar enough to those we observe for Nafion that their use as conductive supports is limited. It may be that future experiments using graphene or other low-contrast, conductive supports will be more productive.

The charge-induced damage mechanism can explain the nonlinear dependence on the dose rate. An alternative explanation may be related to formation of gas in the sample. We speculate that, at some dose rate, evolution of the gas in the film becomes so high that the film rapidly explodes, causing extremely high mass loss. Lower dose rates still create the volatile molecules but those molecules can diffuse to the surface and escape to the vacuum without destroying the film. Slowly escaping gas consists mainly of small molecules and is rich in fluorine, allowing the sample to achieve a more stable chemical configuration.

Electron beam irradiation changes the Nafion composition making it more plastic. We have observed flow of material that smoothes nonuniformities with sizes more than ten nanometers. A related illustration of plasticization is the closing of holes under surface tension. Positioning a sharply focused beam on the surface of Nafion rapidly drills a hole in the film. Spreading the beam then allows one to clearly see the hole and observe that it slowly closes under irradiation. Similar behavior may sometimes be observed on samples of vitreous frozen water. Our observations show that plasticization is not an effect of increased specimen temperature because the same behavior is observed both at room temperature and with cryo-preserved samples.

Our diffraction experiments differ from the observation of single crystals with micron size that showed hexagonal symmetry [4]. The crystals of polytetrafluoroethylene that we observe in virgin Nafion have much smaller sizes, (based on the uniformity of the diffraction ring they are significantly smaller than 10 nm). We do observe growth of single crystals to lateral sizes of about 200 nm under irradiation, which is comparable to the previous results, but no hexagonal symmetry was observed.

## 3. Experimental Section

A 5% Nafion 117 solution in a mixture of lower aliphatic alcohols and water was purchased from Sigma-Aldrich. Electron beam-transparent films were prepared by spin-casting on a silicon wafer or by dip casting. For dip casting, the solution was further diluted to 1 percent Nafion and deposited on a copper TEM grid with a lacey or continuous carbon support film. Excess solution was wiped off with filter paper and the sample was left to air dry. For ion exchange with copper, the sample on a copper EM grid was held in a high humidity atmosphere at 50 °C for about 3 days. We found that such treatment was sufficient for the copper from the grid to diffuse into the polyelectrolyte as evidenced by EELS analysis. For lead exchange, a small piece of lead metal was added to the Nafion solution for about 5 min at room temperature. Longer exposure to lead caused strong oxidation of the lead and opaque precipitates in solution.

Bright Field Electron Microscopy investigations were performed on a Philips CM-200 FEG operated at 200 keV equipped with Gatan Ultrascan CCD detector and an FEI Titan 80–300 operated at 300 keV and equipped with Gatan K2 camera operated in an electron counting mode. Diffraction experiments were performed on the CM200. The EELS investigation was performed on an FEI monochromated F20 UT Tecnai at 200 keV equipped with Gatan GIF. For the cryo experiments samples were either cooled inside of the microscope or saturated with water vapor inside of an FEI Vitrobot, plunged into liquid ethane and transferred to the microscope at liquid nitrogen temperature using a cryo-stage. Data analysis was performed in Gatan DigitalMicrograph.

## 4. Conclusions

The images of Nafion we have obtained in this work show strong effects of electron beam-induced damage and help explain differences observed in various EM investigations of Nafion. Lack of control over the dose results in variations in damage and appearance of the membrane. At elevated dose rates the membrane is damaged even before the first image is collected, and assessing the extent of damage is not always straightforward. Dose rate may be the most important parameter responsible for the discrepancy in experimental results. In contrast to the claims in most of the published work that ion exchange enhances the contrast in Nafion, we observe no effect on contrast in undamaged material. However beam damage of ion-exchanged material clearly induces phase separation and produces features with high levels of contrast.

The low level of contrast we observe in low dose images is a good representation of Nafion structure. In dry material stronger scattering from sulfur is balanced by low density of the ether chains compared to the high density of polytetrafluoroethylene. Saturation of the material with water does not create a separate water phase but distributes water over the large hydrophilic phase which then has low contrast similar to the dry hydrophilic phase.

Damage of Nafion by the electron beam should be a major concern in ongoing studies of Nafion. Both direct ionization damage by the beam and the resulting specimen charging contribute to damage effects. One of the most promising methods for damage reduction is using highly a conductive substrate that reduces the charging. While using a carbon support has limited benefits, application of better substrates such as graphene may prove to be useful.

## Supplementary Files

Supplementary File 1 Supplementary Information (PDF, 868 KB)
