# Supplementary material for: Insights on the Study of Nafion Nanoscale Morphology by Transmission Electron Microscopy
Source: Membranes (Basel). 2013 Dec 16;3(4):424–39. doi: 10.3390/membranes3040424 (PMC4021952; doi:10.3390/membranes3040424)
Supplement: Supplementary File 1 — Supplementary Information (PDF, 868 KB) [file membranes-03-00424-s001.pdf]

Supplementary Information

Figure S1. Bright field TEM images of acidic Nafion. (**a**,**b**) images collected at room temperature with 500 e/nm^2 at rate 500 e/nm^2 s; (**a**) from initially undamaged sample; (**b**) after 10000 e/nm^2; (**c**,**d**) images collected at cryo-temperature with 300 e/nm^2 at rate 300 e/nm^2 s; (**c**) from initially undamaged sample; (**d**) after 5000 e/nm^2.

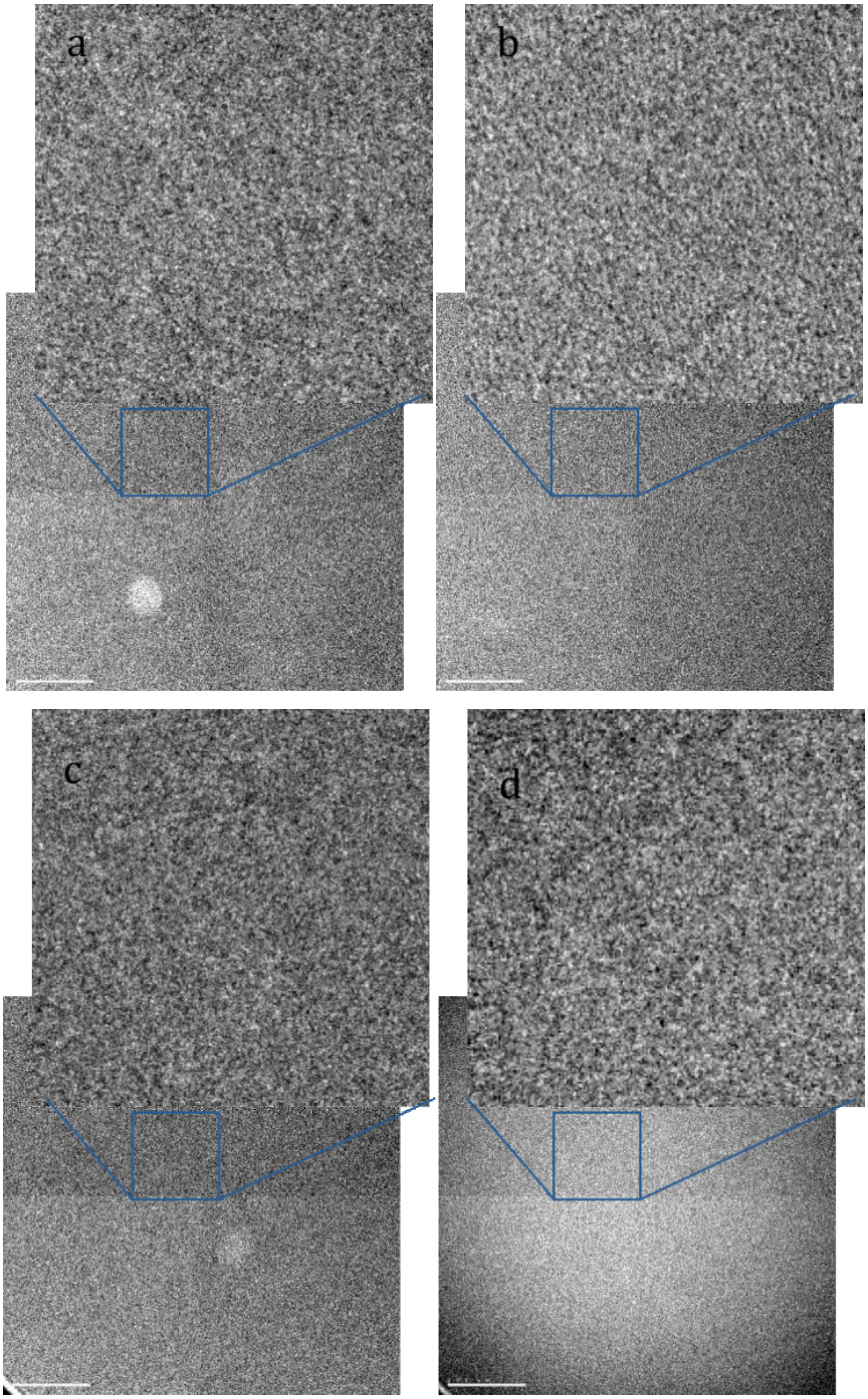

Figure S2. Artifacts in Nafion caused by extended beam irradiation. Sample was exposed to the dose rate of 10000 e/nm^2 s for one minute, resulting in small mass loss after initial thinning but the appearance of substantial phase separation.

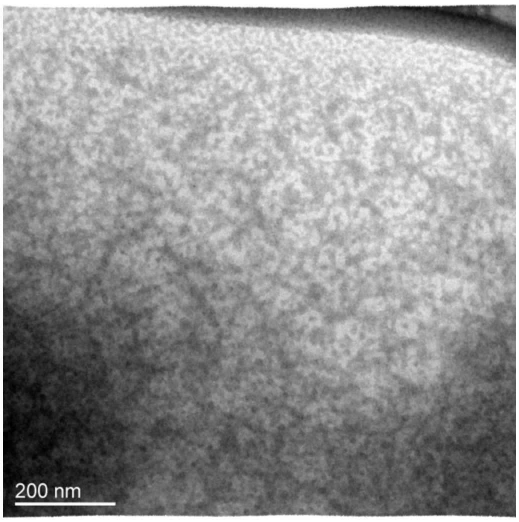

© 2013 by the authors; licensee MDPI, Basel, Switzerland. This article is an open access article distributed under the terms and conditions of the Creative Commons Attribution license (http://creativecommons.org/licenses/by/3.0/).
